# Mobilome of Environmental Isolates of *Clostridioides difficile*

**DOI:** 10.3390/antibiotics14070678

**Published:** 2025-07-04

**Authors:** Khald Blau, Claudia Gallert

**Affiliations:** 1 Department of Microbiology-Biotechnology, Faculty of Technology, University of Applied Sciences Emden/Leer, Constantiaplatz 4, 26723 Emden, Germany; khald.blau@gu.edu.ly; 2 Department of Botany, Faculty of Sciences, University of Gharyan, Gharyan P.O. Box 81006, Libya

**Keywords:** *Clostridioides difficile*, antimicrobial resistance, transposons, plasmids, mobile genetic elements

## Abstract

Background/Objectives: *Clostridioides difficile* is a “One Health” pathogen and a cause of antibiotics-associated diarrhea and pseudomembranous colitis. Mobile genetic elements (MGEs) have been documented in the genomes of clinical *C. difficile* strains; however, the presence of MGEs in environmental strains remains poorly characterized. Thus, the present study was conducted with the objective of identifying the prevalence of MGEs, including mobilizable transposons (MTns), conjugative transposons (CTns), plasmids, and insertion sequences, in whole genome sequences (WGSs) of environmental *C. difficile* isolates. Methods: The analysis of MGEs was conducted using 166 WGSs obtained from *C. difficile* strains isolated from various environmental sources contaminated with feces. The MGEs were identified using bioinformatic tools. Results: A total of 48.2% (80/166) of the studied genomes were identified to harbor nine transposons, including Tn*916*, Tn*6194*-like, Tn*5397*, Tn*6215*, Tn*4001*, Tn*6073*, Tn*6110*, Tn*6107*, or Tn*5801*-like. The majority of MTns and CTns could be found within *C. difficile* sequence types ST11, ST3, and ST35. The results demonstrated close genetic relatedness among the studied genomes, the array of antimicrobial resistance (AMR) genes, such as *tetM*, *ermB*, and *aac(6′)-aph(2″)*, and the presence of CTns. Furthermore, the analysis revealed that 24.7% (41/166) of the genome sequences of isolates were associated with various predominant plasmid groups, including pCD6, pCD-ECE4-6, pCD-WTSI1-4, pCDBI1, and pCd1_3, which belonged to 16 different sequence types. Furthermore, several plasmids were identified as harboring the prophage phiCDHM19. Conclusions: The results of the current study suggest that the identified plasmids are abundant and may encode functions that are relevant to *C. difficile* physiology. The genomes of *C. difficile* strains examined contain closely related CTns, suggesting that horizontal transfer of AMR is important in this species or other bacterial species. Further research is required to ascertain the effect of these genetic elements and their transferability on the biology of *C. difficile*.

## 1. Introduction

*Clostridioides difficile* is a Gram-positive, anaerobic, spore-forming bacterium that is a significant cause of enteric disease in humans and animals [1]. *C. difficile* infection (CDI) depends on the production of toxins A and B, which induce symptoms ranging from mild to severe diarrhea and can result in potentially fatal pseudomembranous colitis [2]. In the past two decades, PCR and sequence-based techniques—effectively equivalent to whole-genome sequencing (WGS)—have advanced our understanding of the genetic diversity, epidemiology, and pathogenicity of *C. difficile* [3]. The organism’s evolutionary plasticity, in response to environmental and anthropogenic pressures, has been shown to play a significant role in the rapid emergence and global dissemination of virulent and resistant clonal lineages [3]. Mobile genetic elements (MGEs) have been shown to profoundly influence the ecology and evolution of organisms, including bacteria. The initial WGS of *C. difficile*, derived from strain CD630, revealed a chromosome of 4.4 Mb and a plasmid, pCD630, of 7881 bp. A high proportion of MGEs, approximately 11% in strain CD630, contributes to the remarkably dynamic and mosaic nature of the *C. difficile* genome. These elements include conjugative and mobilizable transposons, insertion sequences (ISs), IStrons (group I introns), *sigK* intervening elements, CRISPR-Cas systems, genomic islands, the ~7 kb plasmid pCD630, and bacteriophages [4,5]. In our previous study, the genome sequences of environmental *C. difficile* isolates utilized in the current study were found to contain several putative prophages, including 372 intact temperate prophages from the *Myoviridae* and *Siphoviridae* families [6].

It is evident that conjugation, transduction, and transformation of MGEs, most notably transposons, among *C. difficile* strains and/or between *C. difficile* and other bacterial species, represent pivotal mechanisms through which *C. difficile* acquires antimicrobial resistance (AMR) genes [7,8]. For instance, it has been demonstrated that Tn*916* can be transferred from *Bacillus subtilis* to *C. difficile* strains [9]. In addition, the transferability of Tn*5397* between *C. difficile* stains and *B. subtilis* [10,11] and *Enterococcus faecalis* [12] was proven. The presence of several conjugative transposons (CTns) and mobilizable transposons (MTns), a significant proportion of which are associated with AMR genes, has been identified in *C. difficile* [3,4,13]. A putative CTn belonging to the Tn*916*-like family of transposons and carrying the *erm*(B) gene has recently been identified in *C. difficile* 2007855, a strain belonging to the hypervirulent PCR-ribotype 027, and which has been designated as Tn*6194* [14,15]. Another CTn, the Tn*6194* element, which is associated with the *erm*B gene, was identified in *C. difficile* PCR-ribotype 001. This element was subsequently transferred into *C. difficile* PCR-ribotypes 009 and 027, as well as into a strain of *Enterococcus faecalis* [16]. This finding could provide further evidence of the transmission risk of AMR between pathogenic bacteria occupying the same human intestinal niche.

Extrachromosomal elements, such as plasmids, have been found to be significant in most bacterial species. *C. difficile* has been found to possess a significant number of plasmids that encode functions which could be relevant to the pathogenesis of CDI. The majority of previously identified plasmids in *C. difficile* strains were cryptic. These plasmids may encode functions that are relevant to *C. difficile* physiology, including putative plasmid replication and partitioning locus [17,18]. To our knowledge, only a limited number of these plasmids have been associated with AMR or virulence genes. For instance, Ramirez-Vargas and Rodrigues reported the presence of conjugative plasmids in association with *tcdB* and *cdtAB* in clinical *C. difficile* isolates belonging to multilocus sequence typing (MLST) clades C-I, 2, and 4 [19,20]. In recent research, a 7 kb plasmid, pCD-METRO, associated with metronidazole resistance, has been identified in both toxigenic and non-toxigenic human and animal *C. difficile* strains [21]. However, in *C. difficile*, AMR genes are mostly found on transposons rather than on plasmids [13].

Although a few studies have investigated MGEs—including transposons, plasmids, and prophages—in clinical isolates of *C. difficile*, the detection of MGEs in environmental *C. difficile* isolates from various environmental sources remains limited. Therefore, in the present study, the WGSs were collected from 166 *C. difficile* isolates obtained from fecally contaminated environmental sources to assess the prevalence of MGEs, including MTns, CTns, ISs, and plasmids. Furthermore, the present study focused on MGEs carrying genes potentially associated with AMR. The association between identified MGEs and sequence typing (ST) was also examined. It was hypothesized that the genetic characterization of environmental *C. difficile* strains would provide further insight into the role of MGEs in the evolution, pathogenicity, and AMR of *C. difficile*.

## 2. Results

### 2.1. Genetic Diversity of MGEs in Environmental C. difficile Isolates

#### 2.1.1. Mobilizable and Conjugative Transposons

The presence of MTns and CTns was screened in the sequenced genomes of 166 environmental *C. difficile* isolates from diverse fecally contaminated environmental sources, including cattle feces, soil, digested-sludge-amended soils, mixed storage cattle manure, horse feces, thermophilic digesters of biowaste or sewage sludge, biogas plants, anaerobic lab-scale bioreactors for thermophilic digestion of sewage sludge, and samples from a wastewater treatment plant (WWTP), located in northwestern Germany [22,23]. Several MTns and CTns were identified through comparison with reference genomes (Tn*5397* (AF333235.1), Tn*916* (KC414929.1), Tn*6194* (HG475346.1), Tn*5801*-like (EU918655.2)), including Tn*916*, Tn*6194*-like, Tn*5397*, Tn*6215*, Tn*4001*, Tn*6073*, Tn*6110,* Tn*6107*, and Tn*5801*-like. MTns and CTns were detected in 48.2% (80/166) of *C. difficile* isolates (Table 1 and Table S1).

**Figure 1:**
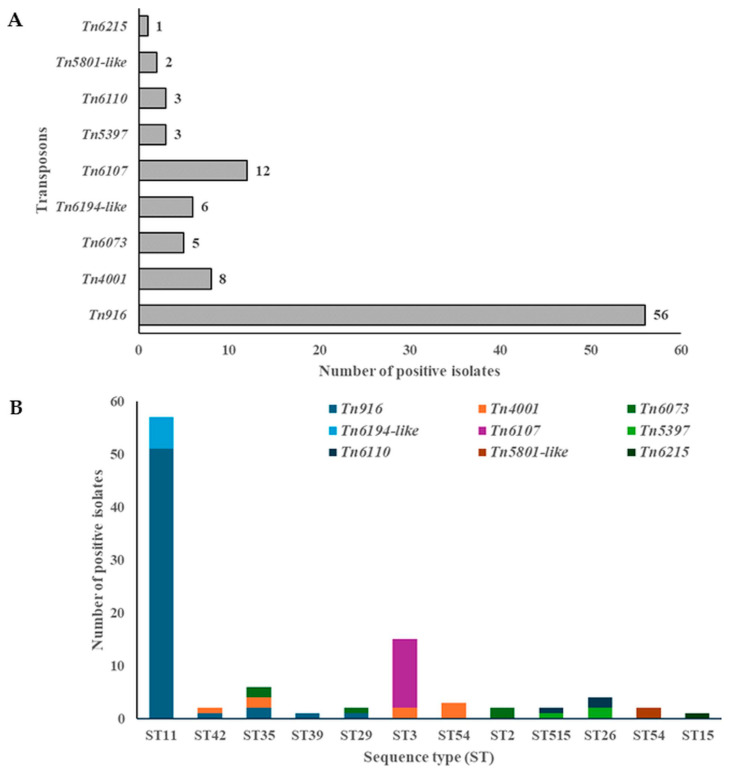
The presence of MTns and CTns (**A**) and their association with sequence types (STs) (**B**) in environmental *C. difficile* isolates.

The majority of MTns and CTns identified were Tn*916*, Tn*6107*, Tn*4001*, Tn*6194*-like, and Tn*6073*, followed by Tn*5397* and Tn*6110*, Tn*5801*-like, and Tn*6215*. These were found in 56 (33.7%), 12 (7.2%), 8 (4.8%), 6 (3.6%), 5 (3%), 3 (1.8%), 2 (1.2%), and 1 (0.6%) isolates, respectively (Figure 1A). To establish a genomic context for the analysis of AMR genes, a screening procedure was employed in which draft genomes were examined for the presence of MTns and CTns. The most prevalent transposons carrying *tetM* were Tn*916* (33.7%, 56/166), Tn*5397* (1.8%, 3/166), and Tn*5801*-like (1.2%, 2/166), which were widely distributed among strains from diverse sources (Table 1 and Table S1). Transposons carrying the *ermB* gene included Tn*6194*-like (3.6%, 6/166) and Tn*6215* (0.6%, 1/166). Tn*6215* was identified exclusively in the RSS11/RT010/ST15 strain (Table 1 and Table S1). Tn*4001* was identified as containing IS*256* elements flanking the *aac(6′)-aph(2″)* gene, which confers resistance to gentamicin, tobramycin, and kanamycin. These elements were verified in eight isolates (4.8%) (Table 1 and Table S1).

Most MTns and CTns were found within ST11 (51 (60.6%)), ST3 (15 (16%)), ST35 (6 (6.4%)), ST26 (4 (4.3%)), and ST54 (3 (3.2%)) (Figure 1B). Among these STs, ST35 demonstrated the highest level of diversity, with three different MTns/CTns identified. The following STs, ST11, ST42, ST3, ST515, and ST26, each exhibited two different MTns/CTns. A single instance of MTns/CTns was identified among the remaining STs (Figure 1B). Tn*5397*, also known as CTn3, was found in 3 out of 166 isolates (1.8%) (TDS118/RT140/ST515, RSS12/RT140/ST26, and RSS52/RT140/ST26). The *tndX* gene, which encodes a large serine recombinase, was identified in all Tn*5397* transposons within the genomes of three strains that have been isolated from a thermophilic digester treating sewage sludge and raw sewage sludge. This recombinase is essential for the insertion and excision of Tn*5397*. All Tn*5397* transposons were found to carry the tetracycline resistance gene *tetM*. Tn*5397* was identified in only two different STs: ST515 and ST26 (Figure 1B).

Tn*916* is a prominent transposon family that has been documented in *C. difficile* strains and is known to carry the *tetM* gene. In the present study, Tn*916* was identified in the majority (33.7%) of environmental *C. difficile* isolates (Table 1), which were recovered from calf feces, raw sewage sludge, digested-sludge-amended soils, and biogas plants (Table 1 and Table S1). The *tetM* and *int* genes, which is responsible for insertion, were observed in all Tn*916* transposons. Tn*916* was predominantly identified in hypervirulent RTs, including RT078, RT126, and RT127, which belong to the ST11 group. Notably, a considerable proportion of environmental *C. difficile* isolates exhibited the presence of multiple Tn*916* transposons, with 1–4 copies per isolate, as documented in Table S1.

**Figure 2:**
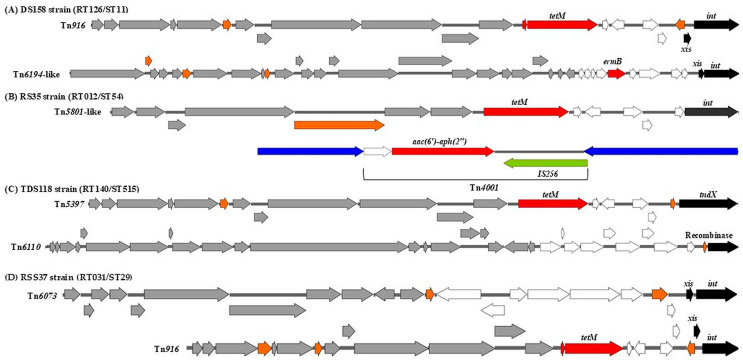
Schematic view of representative transposons in a selection of *C. difficile* strains. Black: recombination genes, white: transcriptional regulation, red: accessory genes (AMR genes), grey: conjugation modules, orange: hypothetical proteins or proteins of unknown function. The schematic of transposons was generated using SnapGene (version 8.0.1).

It was found that a significant proportion of environmental *C. difficile* strains are characterized by the presence of multiple MTns and/or CTns. For instance, Tn*916* and Tn*6194*-like were identified within the same chromosomal genome of the strain DS158/RT126/ST11, which carried *tetM* and *ermB* genes, encoding resistance to tetracycline and aminoglycosides, respectively (Figure 2A). Furthermore, the strain RS35/RT012/ST54 was found to carry the transposons Tn*5801*-like and Tn*4001*, which are associated with *tetM* and *aac(6′)-aph(2″*) resistance genes, respectively (Figure 2B). In addition, strains TDS118/RT140/ST515 and RSS37/RT031/ST29 carried two different transposons (Tn*5397* and Tn*6110*, Tn*916* and Tn*6073*), respectively (Figure 2C,D).

#### 2.1.2. Plasmids

**Figure 3:**
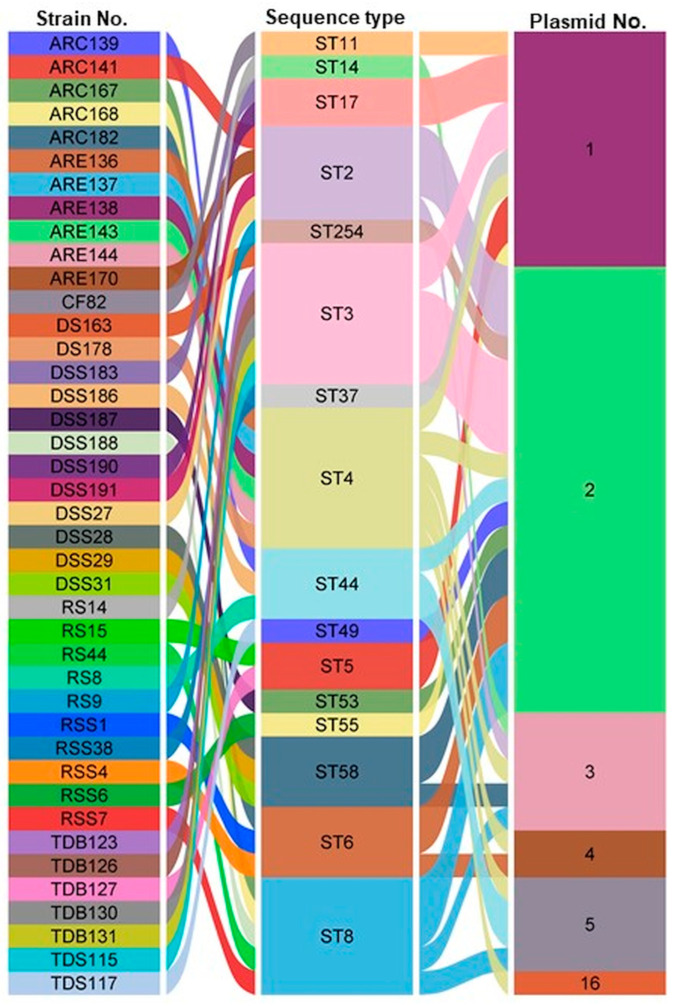
An alluvial diagram representing, from right to left, the number of identified plasmids and their association with STs and *C. difficile* strains isolated from different environmental sources. The width of each connection is proportional to the number of positive isolates. RSS: raw sewage sludge, RS: raw sewage, DSS: digested sewage sludge, CF: calf feces, ARC: anaerobic lab-scale bioreactors treating sewage sludge/control, ARE: anaerobic lab-scale bioreactors treating sewage sludge/experiment, DS: digested-sewage-sludge-amended soils, TDS: thermophilic digester treating sewage sludge, TDB: thermophilic digester treating biowaste. The alluvial diagram was generated using SRplot [24].

It is hypothesized that this study is the first to demonstrate the presence of a substantial number of plasmids in the genomes of *C. difficile* isolates obtained from diverse fecally contaminated environmental sources. Plasmids exhibiting nearly 80–100% coverage were identified in 41 out of 166 isolates, corresponding to 24.7%, and ranged in size from 801 bp to 120,098 bp across 16 distinct ST patterns (Figure 3 and Table S2). Most isolates carried between one and sixteen plasmids in their genomes. The most common plasmid counts per isolate were 2, 1, 3, and 5, found in 19, 10, 5, and 4 isolates, respectively. In contrast, two isolates carried four plasmids, and one isolate was found to harbor sixteen plasmids (Figure 3).

The majority of plasmids were identified in the genome sequences of strains belonging to ST4, ST3, and ST8, with seven, six, and five isolates, respectively. Meanwhile, plasmids of the specified types were observed in three isolates each from ST44, ST2, ST58, and ST6, while the other STs occurred at lower frequencies, being represented by only one or two isolates each (Figure 3). This study also demonstrated that 7.2% (12/166) of environmental *C. difficile* strains carry a plasmid belonging to the pCD6 family (Table S2). The majority of pCD6 plasmids were found in isolates belonging to ST4, ST2, and ST8, which were obtained from DSS, ARC and ARE, and TDB and TDS (Figure 3 and Table S2).

Plasmids belonging to the pCD-WTSI subfamilies were identified in 12.04% (20/166) of environmental *C. difficile* isolates (Figure 3 and Table S2). The pCD-WTSI subfamilies were distributed across a range of STs, including ST58, ST44, ST3, ST6, ST49, ST14, and ST8 (Figure 3 and Table S2). Within the pCD-ECE families, four plasmids were identified in environmental *C. difficile* strains belonging to the pCD-ECE4 family (2.4%), whereas the pCD-ECE5 and pCD-ECE6 families were each identified in a single isolate. The pCD-ECE1, pCD-ECE2, and pCD-ECE3 plasmids were not detected. The maximum number of ECEs documented in a single isolate was two. The majority of ECEs were observed in isolates belonging to ST58, ST14, and ST8 (Table S2). The present study has revealed that plasmids of the identified types are present in the genomes of *C. difficile* isolates recovered from various fecally contaminated environmental sources, with the exception of samples derived from activated sewage sludge (ASS) and biogas plants (BPs) (Figure 3 and Table S2).

### 2.2. Phylogenetic Analysis of Identified Plasmids in Environmental C. difficile Genome Sequences

**Figure 4:**
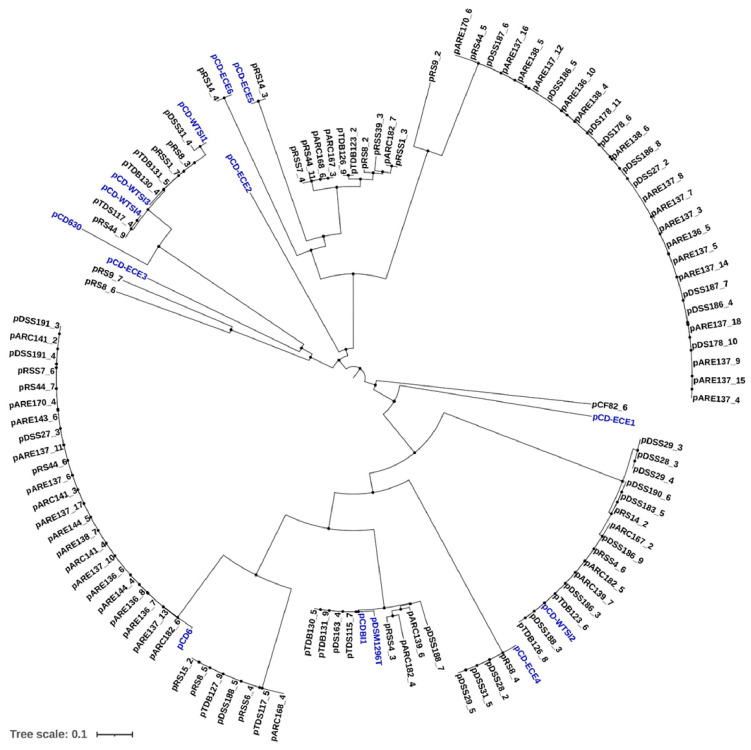
Phylogenetic analysis of identified plasmids in environmental *C. difficile* strains. Reference plasmids (blue), pCD-ECE6 (LR594546.1), pCD-ECE4 (LR594545.1), pCD-WTSI2 (NZ_JADKQL030000003.1), pCD6 (NC_005326.1), pCD-WTSI4 (NZ_MG019962.1), pCD-WTSI3 (NZ_MG019961.1), pCD-ECE5 (LR594541.1), pCD-WTSI1 (NZ_MG019959.1), pCDI1 (NC_017176.1), pDSM1296 (NZ_CP011969.1), pCD-ECE1 (NZ_LR594544.1), pCD-ECE2 (NZ_LR594542.1), pCD-ECE3 (NZ_LR594543.1), and pCD630 (NC_008226.2).

A phylogenetic tree was constructed using the complete sequences of both the identified plasmids and reference plasmids. Potential plasmids extracted from the genomes of environmental strains were clustered based on their average nucleotide identity (ANI) with available reference sequences. Among the plasmids identified in this study, 23 fall within a distinct pCD6 cluster (Figure 4). Based on phylogenetic analysis, a significant proportion (4.8%, 8/166) of environmental strains carry cryptic 45–48 kb plasmids belonging to the pDLL3026 family. Additionally, four plasmids were found to cluster distinctly within the pCDBI1 and pDSM1296 plasmids (Figure 4).

It is noteworthy that the identified plasmids carrying phage-related functions were distributed into two distinct clusters. One of these clusters exhibited 100% sequence identity with the reference plasmids pCDBI1 and pDSM1296 and included plasmids, such as pTDB130_5, pTDB131_9, pDS163_4, and pTDS115_7 (Figure 4). Moreover, these plasmids, which belong to the pDLL3026 family, were found exclusively in ST3 isolates (Table S2). The second cluster comprises several plasmids identified in the current study, including pRSS7_4, pRS44_11, pARC167_3, pARC168_6, pTDB123_2, pTDB126_9, pRSS39_3, pRS8_2, pARC182_7, and pRSS1_3 (Figure 4). These plasmids are hypothesized to have arisen through recombination with bacteriophages, despite retaining key plasmid features, such as a replication initiation protein (RepB).

The potential plasmids identified in this study were also found to be similar to particular clusters of pCD-ECE4, pCD-ECE5, or pCD-ECE6 plasmids, with four corresponding to pCD-ECE4 and one each to pCD-ECE5 and pCD-ECE6 (Figure 4). Plasmids pRS9_7, pRS9_2, and pRS8_6 were found to be closely related, exhibiting 100% identity and over 98% coverage with *Enterococcus faecium* plasmids (Figure 4 and Table S2). Furthermore, an additional 22 plasmids demonstrating 99–100% sequence identity and coverage with the pCD-WTSI subfamilies were also identified (Figure 4 and Table S2).

A total of four identified plasmids, pRS15_2, pRS8_5, pDSS188_5, and pARC168_4, were closely related, showing 100% sequence identity and coverage with the pCD-ECE6 plasmid (Table S2). However, these plasmids did not cluster with the reference plasmid (Figure 4). Instead, they formed a cluster with three other previously identified plasmids (pTDB127_9, pRSS6_4, and pTDS117_5), which were identical to the reference plasmid pAR1090_2 (Table S2). Finally, 27 plasmids—ranging from pARE170_6 to pARE137_4 (Figure 4)—were found to be identical to the reference plasmids pCd1_3, pCd11_5, pCd8, or pCd5_4 (Table S2). Notably, these reference plasmids were all isolated from the same source.

### 2.3. Comparison of the Identified Plasmids with Reference Plasmids

**Figure 5:**
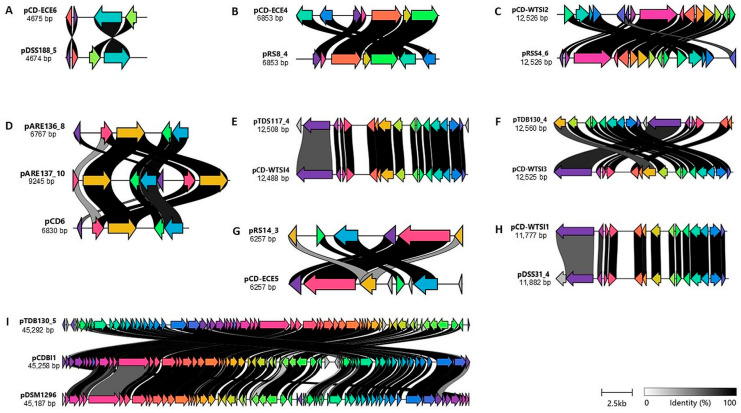
Comparison of representative identified plasmids with reference plasmids. Within the panels, the presence of colored arrows indicates the presence of similar genes; links are drawn between similar genes on neighboring clusters and shaded based on sequence identity (0% white, 100% black, identity threshold for visualization 0.30). (**A**). Comparison of pDSS188_5 with pCD-ECE6 (LR594546.1). (**B**). Comparison of pRS8_4 with pCD-ECE4 (LR594545.1). (**C**). Comparison of pRSS4_6 with pCD-WTSI2 (NZ_JADKQL030000003.1). (**D**). Comparison of pARE137_10 and pARE136_8 with pCD6 (NC_005326.1). (**E**). Comparison of pDTS117_4 with pCD-WTSI4 (NZ_MG019962.1). (**F**). Comparison of pTDB130_4 with pCD-WTSI3 (NZ_MG019961.1). (**G**). Comparison of pRS14_3 with pCD-ECE5 (LR594541.1). (**H**). Comparison of pDSS31_4 with pCD-WTSI1 (NZ_MG019959.1). (**I**). Comparison of pTDB130_5 with pCDI1 (NC_017176.1) and pDSM1296 (NZ_CP011969.1). The image has been derived from a visualization using clinker [25].

The assignment of the representative identified plasmids to the reference plasmids is preliminary and based on regions of similarity to the reference plasmid sequences. To gain further insight into the relatedness of the plasmids, nucleotide alignments were visualized using clinker [25]. Several observations emerged from these analyses. Firstly, the pTDS117_4 plasmid appears to contain a 17-gene insertion compared to the pCD-WTSI4 reference sequence. The ORF1 and ORF17 regions of pTDS117_4 appear to be non-conserved in the reference plasmid pCD-WTSI4 (Figure 5E). ORF1 of pTDS117_4 encodes a DEAD/DEAH box helicase, while ORF17 encodes a non-functional (hypothetical) protein. Secondly, the ORF4, ORF6, and ORF8 regions of the reference pCD-ECE5 plasmid appear to be non-conserved in the pRS14_3 plasmid (Figure 5G). These three ORFs in the pCD-ECE5 reference sequence encode non-functional proteins. Thirdly, the pDSS31_4 plasmid contains a conserved region but also includes one insertion compared to the reference plasmid pCD-WTSI4 (Figure 5H). ORF1 of pDSS31_4 is hypothesized to encode an additional DEAD/DEAH box helicase. Finally, the plasmid pARE137_10 contains a 7-gene insertion compared to the pCD6 reference sequence and the pARE136_8 plasmid sequence. ORF1 of pARE137_10 encodes a hypothetical protein of unknown function, while ORF7 is hypothesized to encode an additional replication protein region, including the *repA* gene, which encodes a 545-amino acid protein (Figure 5D).

It is probable that the non-conjugative cryptic plasmids belong to the pDLL3026 plasmid family, which includes pCDBI1, pDSM1296, and other plasmids identified in the present study, such as pTDB130_5 (Figure 5I). These plasmids contain a RepB, genes involved in transcriptional regulation, and members of the ParM/StbA family, as well as several phage-related functions. Furthermore, the annotation of the pTDB130_5 plasmid revealed the presence of 69 ORFs, whereas the reference plasmids pCDBI1 and pDSM1296 contain 66 and 67 ORFs, respectively. The predicted functions of these ORFs were categorized into seven groups: plasmid maintenance, DNA replication and repair, restriction endonuclease and DNA modification, transcriptional regulation, recombination, hypothetical proteins and proteins of unknown function, and phage or phage-related functions (Figure 5I).

### 2.4. The Genome Organization of Identified Representative Cryptic Plasmids of C. difficile

**Figure 6:**
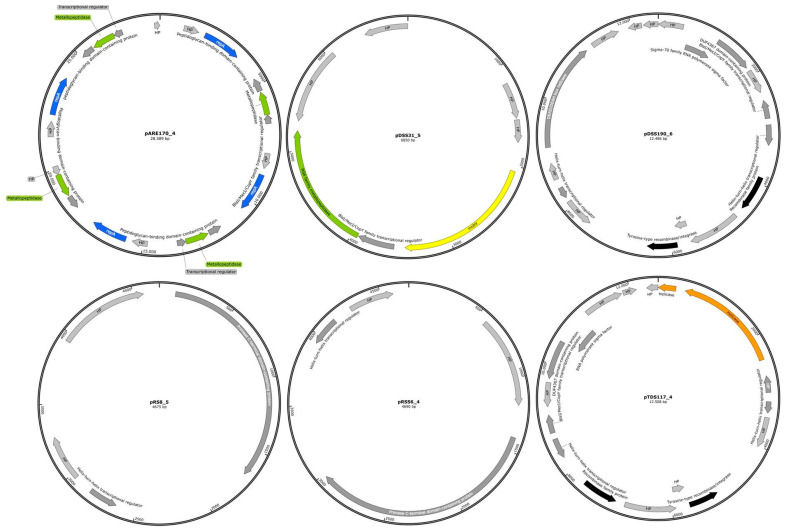
Genome organization of representative cryptic *C. difficile* plasmids. Predicted CDSs are marked with arrows and colors that indicate functional assignments: plasmid maintenance (blue), DNA replication and repair (orange), restriction endonuclease and DNA modification (green), transcriptional regulation (dark grey), recombination (black), hypothetical proteins and proteins of unknown function (light grey), and conjugation modules (yellow). The genome maps of representative identified plasmids were generated using SnapGene (version 8.0.1).

All plasmids identified in the studied genome sequences of different environmental *C. difficile* strains were found to be cryptic. Therefore, representative identified plasmid genomes are displayed schematically in Figure 6. The plasmids pRS8_5 and pRSS6_4 contained only four annotated genes, while pDSS31_5, pTDS117_4, pDSS190_6, and pARE170_4 contained 7, 17, 17, and 21 genes, respectively. The majority of these genes encode proteins involved in transcriptional regulation, as well as proteins of hypothetical or unknown function. In many bacterial species, plasmids play a significant role in AMR, often mediating the transmission of resistance genes. However, in *C. difficile*, AMR genes are predominantly located on transposons rather than on plasmids [13]. Additionally, a total of 17 identified plasmids were found to carry phage-related genes. These genes were specifically associated with the intact prophage phiCDHM19. One plasmid, pARC182_4 (RT005/ST6), was found to be linked to the incomplete prophage phiCDHM11 (Table S3). Reference plasmids—including pCDBI1, p830101_1, pCd11_3, pCd10_2, pJ21_1, pDSM1296, and unnamed 1_CD4 that are identical to the identified plasmids—were screened for the presence of prophage phiCDHM19. The results indicated that all reference plasmids harbored the same prophage as those identified in the environmental *C. difficile* genomes.

## 3. Discussion

It is evident that essential MGEs and integrative conjugative elements (ICEs) play a crucial role in horizontal gene transfer (HGT), facilitating increased genetic diversity and the acquisition of exogenous genetic material [14]. Transposons, including Tn*916*, Tn*5397*, Tn*5398*, and Tn*4453*, have been widely distributed across diverse STs of *C. difficile* isolates [13]. In this study, nine types of MTns or CTns could be identified in the genome sequences of environmental *C. difficile* isolates, including Tn*916*, Tn*6194*-like, Tn*5397*, Tn*6215*, Tn*4001*, Tn*6073*, Tn*6110*, Tn*6107*, and Tn*5801*-like. These transposons were identified in 11 distinct STs, the majority of which belonged to hypervirulent RTs, including RT126, RT127, and RT78, which were found to be associated with the ST11 group.

The most commonly detected transposons in environmental *C. difficile* genomes were Tn*916*, Tn*6107,* Tn*4001*, and Tn*6194*-like, with Tn*916* being the most prevalent, present in 33.7% of isolates. Notably, Tn*916* was predominantly linked to hypervirulent RTs, such as RT126, RT127, and RT078, which are all part of the ST11 lineage and carry the *tetM* gene. The acquisition of tetracycline resistance in *C. difficile* strains has previously been linked to transposons including Tn*5397*, the Tn*916*-like family, and Tn*6164* [26]. The findings of the present study align with previous research showing Tn*916* (53.7%) and Tn*5397* (31.48%) as the most prevalent *tetM*-carrying transposons among environmental, animal, and clinical strains in China [27]. Furthermore, *tetM* associated with Tn*916* has also been identified in both toxigenic and non-toxigenic *C. difficile* strains from Southeast Asia [28]. Dingle et al. reported that 76.5% of agriculture-associated *C. difficile* RT078 strains were positive for the *tetM* gene [29]. Overall, Tn*5397* and Tn*916*-like transposons are the predominant genetic elements mediating tetracycline resistance through the *tetM* gene [7,30].

In addition to Tn*916*-like elements, *tetM* was linked to Tn*5397* and Tn*5801*-like transposons, detected in three and two isolates, respectively. This highlights the potential role of CTns in driving the evolution and widespread dissemination of *tetM* among both toxigenic and non-toxigenic *C. difficile* strains and possibly to other enteric pathogens. For instance, it has been demonstrated that Tn*916*, originally identified in *C. difficile*, can be transferred to *Bacillus subtilis* [9]. Tn*5397*, formerly known as CTn*3*, was first identified in *C. difficile* strain 630. This 21 kb element closely resembles the Tn*916*-like family [4], which is the paradigm of this MGE family. The transferability of Tn*5397* has been demonstrated between *C. difficile* stains and *B. subtilis* [10,11] and *Enterococcus faecalis* [12]. Vries et al. reported that Tn*5801*-like genomic islands (GIs) are widespread in tetracycline-resistant *Staphylococcus pseudintermedius*, as well as in other Gram-positive species [31]. While Tn*5801* shares several ORFs with Tn*916*, it differs by containing a unique integrase gene (*int*_Tn*5801*_) instead of the *xis*_Tn*916*_*/int*_Tn*916*_ excisionase/integrase system found in Tn*916*. Tn*916* was detected in *C. difficile* isolates from calf feces and WWTP samples. Agricultural use of tetracyclines likely drives selection pressure and, coupled with AMR spread via the food chain, facilitates the persistence of resistant *C. difficile* strains in both clinical and environmental contexts. Resistance to macrolide–lincosamide–streptogramin B (MLS_B_) antimicrobials in *C. difficile* strains is predominantly mediated by the *ermB* gene. In the present study, transposons carrying the *ermB* gene were identified, including Tn*6194*-like (3.6%) and Tn*6215* (0.6%). The Tn*6194*-like element was detected in the genome sequences of RT126 and RT078 strains belonging to the ST11 group, while Tn*6215* was identified in an RT010/ST15 strain. A recent study involving environmental, animal, and clinical isolates reported that *ermB*-carrying transposons could be classified into four categories: Tn*6189* (9.0%), Tn*6194* (5.3%), Tn*6215* (9.0%), and Tn*6218* (64.0%), based on their distribution among different origins [27]. Additionally, Imwattana et al. demonstrated that both toxigenic and non-toxigenic *C. difficile* strains from Southeast Asia harbor the *ermB* gene on various transposons, such as Tn*6218*, Tn*6189*, Tn*6194*-like, and Tn*6215* [28]. Further analysis of publicly available genomes of *ermB*-positive *C. difficile* strains revealed that the most common *ermB*-positive transposon is Tn*6194* (44.4%), followed by Tn*6189* (23.9%) and Tn*6218* (12.2%), among 1775 strains [32]. Wasels et al. showed that the *ermB*-carrying Tn*6194*-like, identified in a *C. difficile* RT001 strain, was capable of horizontal transfer of AMR not only within *C. difficile* strains (RT009 and RT027) but also between *C. difficile* and *Enterococcus faecalis* [16].

The transposon Tn*4001* was found to contain a single copy of the IS*256* element, which encodes a bifunctional aminoglycoside-modifying enzyme with both acetyltransferase and phosphotransferase activity—specifically, *aac(6′)-aph(2″)*—in 4.8% of the 166 *C. difficile* strains analyzed. As demonstrated by Ramírez-Vargas et al., a sequence copy of the composite Tn*4001* could be detected in human *C. difficile* isolates of the NAP_CR1_/RT012/ST54 genotype. This sequence was found to be inserted within a novel putative MTn, which also carried the *aac(6′)-aph(2″)* gene [33]. The composite transposon Tn*4001*, which consists of two flanking IS*256* elements, was originally identified in *Staphylococcus aureus* [34].

In addition, three other transposons, Tn*6073*, Tn*6107* (CTn5-like element), and Tn*6110*, were detected in 3.0%, 7.2%, and 1.8% of *C. difficile* isolates, respectively. Brouwer et al. reported the presence of Tn*6073*, Tn*6107*, and Tn*6110* in *C. difficile* strains QCD-23M63, QCD-23M63, and QCD-66C26, respectively [4]. The accessory modules of these transposons encode a diverse set of genes, including those for a predicted N-terminal hydrolase, a sigma factor, putative transcriptional regulators, and ABC transporters, all of which may play roles in facilitating the adaptation and persistence of *C. difficile* across diverse environmental niches. 

Among the 166 *C. difficile* genomes analyzed in the present study, 41 (24.7%) were found to harbor potential plasmids, distributed across 16 distinct STs. These plasmids were predominantly classified into major groups such as pCD6, pCD-ECE4–6, pCD-WTSI1–4, pCDBI1, and pCd1_3, highlighting the widespread occurrence and diversity of plasmids within the species. Previous studies have demonstrated that *C. difficile* strains from diverse geographic regions harbor plasmids of varying sizes [35]. For example, Dost et al. reported plasmid presence in 87.7% (471/537) of a *C. difficile* genome collection belonging to ST8/RT002, with the most prevalent plasmids being pCDBI1, pAK2, pCDT4, pCD116-S, and plasmid 1 from *C. difficile* strain FDAARGOS_267 [36]. In the present study, it was also observed that individual isolates can carry multiple plasmids from specific families. This observation aligns with prior in silico analyses of publicly available genome data, which have indicated the co-occurrence of multiple plasmids within a single isolate and suggested that certain plasmid families may be compatible and capable of co-existing within the same host strain [18,37]. Roseboom et al. demonstrated that a human clinical isolate carried three plasmids from three different plasmid families, including pCD-ECE1, pCDWTSI1, and pCD630 [38].

The present study investigated the prevalence of a specific plasmid, pCD6. A total of 7.2% of *C. difficile* strains from different environmental sources were found to carry this plasmid. pCD6 remains the only *C. difficile* plasmid that has been fully characterized. Its 6.8 kb nucleotide sequence was originally identified during attempts to establish genetic manipulation methods in strain CD6 [39]. Moreover, the copy number of the pCD6 plasmid ranged between 4 and 10 [40]. The pCD6 replicons are characterized by encoding a RepA that shows significant similarity to the RepA protein of the *C. perfringens* plasmid pIP404 [41]. A comprehensive sequencing study conducted at a major Australian hospital revealed the presence of various plasmids, including pCD6, pCDBI1, pDLL3026, and pCD630, in approximately 27% of *C. difficile* strains analyzed (19 out of 71). Notably, pCD6 was identified in 12 of these strains, constituting the most prevalent plasmid detected [42].

Approximately 4.8% of environmental *C. difficile* strains were found to harbor cryptic 45–48 kb plasmids, classified within the pDLL3026 family. This group includes pCDBI1, which carries prophage sequences homologous to the intact temperate phage phiCDHM19, suggesting a potential role in HGT or lysogenic conversion. In our previous study, 17% of intact phiCDHM19 prophages were identified within contigs of environmental *C. difficile* genome sequences [6]. This finding is consistent with a prior study in which 4.9% of *C. difficile* isolates were found to carry cryptic plasmids ranging from 42 to 47 kb. These plasmids are believed to have arisen through recombination with bacteriophages but still retain essential plasmid features [17]. Furthermore, the pDLL3026 plasmid was found to contain multiple putative bacteriophages, including temperate phages from the *Myoviridae* family and one intact temperate phage from the *Siphoviridae* family [17].

In the present study, pCD-ECE family elements were identified in 3.6% of the 166 *C. difficile* genome sequences, with 1–2 elements per strain from different subfamilies. In contrast, Hornung et al. reported that approximately 13% of *C. difficile* sequence data contained ECEs, with 1–6 elements per strain [18]. In this study, most ECEs were affiliated with various STs, such as ST58, ST14, ST44, ST8, and ST5. Conversely, analysis of publicly available *C. difficile* genomes revealed that the majority of ECEs were associated with ST1, ST11, and ST37 [18].

In addition to the many new findings, the currently existing limitations of this investigation should also be mentioned. First, a comparative genomic analysis of environmental and clinical *C. difficile* strains from the same geographic region could not be performed, as only a limited number of human isolates were available. This constraint limits the ability to assess potential transmission dynamics or genetic overlap between environmental and clinical populations. Second, it is evident that research in this area is limited, with no studies to date investigating the transferability of AMR-associated MGEs both within *C. difficile* populations and between *C. difficile* and other pathogenic bacteria originating from diverse environmental niches.

## 4. Materials and Methods

### 4.1. Isolation and Identification of C. difficile

*C. difficile* isolates utilized in the present study were recovered from 81 fecally contaminated environmental samples collected from March 2021 to June 2022, including WWTP samples, calf feces, cattle-feces-contaminated soil, thermophilic digesters treating biowaste and sewage sludge, and digested-sewage-sludge-amended soils, as previously described [22]. Briefly, environmental samples were inoculated in *C. difficile* selective (CD) broth supplemented with 12 mg/L norfloxacin (Sigma-Aldrich Chemie GmbH, Munich, Germany) and 32 mg/L moxalactam (Biomol GmbH, Hamburg, Germany) and 0.1% sodium taurocholate (Carl Roth GmbH & Co. KG, Karlsruhe, Germany) for spore germination. All inoculated CD broths were prepared anaerobically in an anaerobic chamber (Coy Laboratory Products, Inc. Los Angeles, CA, USA) and flushed with a gas mixture (80% N_2_ and 20% CO_2_). All inoculated CD broths were incubated at 37 °C for 7–10 days. Each incubated CD broth was then mixed with an equal volume of absolute alcohol (1:1) and incubated at room temperature for 50–60 min. The mixtures were then centrifuged at 4000 rpm for 10 min and the supernatant was discarded. The pellet was resuspended in 1 phosphate-buffered saline (PBS) and plated on *Clostridium difficile* agar (CDA, Fisher Scientific GmbH, Schwerte, Germany) supplemented with 7% defibrinated horse blood (Fisher Scientific GmbH, Schwerte, Germany), 12 mg/mL norfloxacin, 32 mg/mL moxalactam, and 0.1% sodium taurocholate. All plates were incubated anaerobically in anaerobic jars (Schuett-Biotec GmbH, Göttingen, Germany) (80% N_2_, 10% H_2_, and 10% CO_2_) at 37 °C for 2–5 days. Selected colonies were evaluated by morphology and confirmed by the Oxoid *C. difficile* latex agglutination test (Fisher Scientific GmbH, Schwerte, Germany). The final confirmation was made by analyzing the specific housekeeping gene, triose phosphate isomerase (*tpi*), as previously described by Leeme et al. [43].

### 4.2. Whole Genome Sequencing and Data Analysis

In total, 166 environmental *C. difficile* isolates were subjected to WGS using the Pacific Biosciences long-read platform Sequel IIe (Pacific Biosciences Inc., Menlo Park, CA, USA) and were subsequently assembled de novo using the SMRT Link software versions 10 and 11 (Pacific Biosciences Inc.) as described recently [44]. The MLSTs (STs) were determined according to the *C. difficile* MLST database of the PubMLST website (https://pubmlst.org/organisms/clostridioides-difficile (accessed on 15 November 2022)). All sequenced contigs were annotated using the rapid annotation using subsystem technology (RAST) web server version 2.0 (https://rast.nmpdr.org/ (accessed on 15 November 2022)). AMR genes were identified in the genome sequences of 166 *C. difficile* strains with the comprehensive antibiotic resistance databases (CARD) version 2 using resistance gene identifier (RGI) (https://card.mcmaster.ca/ (accessed on 11 April 2023)), BacAnt [45], ResFinder 4.1 (https://cge.food.dtu.dk/services/ResFinder/ (accessed on 11 April 2023)) [46], ARG-ANNOT [47], and Vrprofile2 [48]. The previously deposited WGS data, including the complete set of contig sequences, were submitted to the National Center for Biotechnology Information (NCBI) GenBank database under BioProject accession number PRJNA1011814 [23]. The accession numbers for the individual plasmid sequences identified in this study are listed in Table S2.

### 4.3. Analysis of MGEs

MTns, CTns, and their association with AMR genes were identified in *C. difficile* genome sequences using web servers, such as BacAnt [45] and Vrprofile2 [48]. These findings were complemented by a BLASTn search of the *C. difficile* genome sequences or transposon sequences available at NCBI (https://www.ncbi.nlm.nih.gov/ (accessed on 24 October 2024)). The MTns and CTns were defined as having >80% nucleotide identity and coverage [49]. To identify plasmids, contigs were screened for plasmids using BLASTn against the NCBI complete plasmid database. The potential plasmids were defined as having ≥70% coverage and ≥80% identity [49]. Prophages were identified using the PHASTEST web server in the identified plasmids which carry phage-related genes. The intact, questionable, and incomplete prophage sequences were defined by score values of >90, 70 to 90, and <70, respectively [50].

### 4.4. Phylogenetic Tree of Identified Plasmids in Environmental C. difficile Strains

The construction of the phylogenetic tree was achieved by employing the complete sequences of the identified and reference plasmids in order to ascertain the genetic relatedness between the identified plasmids and well-known reference plasmids, such as pCD-ECE6 (LR594546.1), pCD-ECE4 (LR594545.1), pCD-WTSI2 (NZ_JADKQL030000003.1), pCD6 (NC_005326.1), pCD-WTSI4 (NZ_MG019962.1), pCD-WTSI3 (NZ_MG019961.1), pCD-ECE5 (LR594541.1), pCD-WTSI1 (NZ_MG019959.1), pCDI1 (NC_017176.1), pDSM1296 (NZ_CP011969.1), pCD-ECE1 (NZ_LR594544.1), pCD-ECE2 (NZ_LR594542.1), pCD-ECE3 (NZ_LR594543.1), and pCD630 (NC_008226.2). The Newick tree was made using the NGPhylogeny.fr web server (https://ngphylogeny.fr/, accessed on 15 February 2025) [51]. Subsequently, the phylogenetic analysis was visualized using the integrative Tree of Life (iTOL) web server.

### 4.5. Comparison of the Identified Plasmids with Reference Plasmids

In the present study, the representatively identified plasmids were compared with the reference plasmids. This is achieved by basing the comparison on regions of similarity with the reference plasmid sequences. In order to gain further insight into the genetic relatedness of the plasmids, nucleotide alignments were visualised using Clinker [25].

## 5. Conclusions

The present study showed that putative MTns and CTns are present in nearly half of the genomes of environmental *C. difficile* strains. Despite the variability exhibited by the accessory regions of the CTns, several distinct types of core elements were identified, including Tn*916*, Tn*5397*, Tn*6194*, and Tn*6215*. These elements were found to be associated with AMR genes that encode tetracycline and aminoglycoside resistance. Furthermore, given that some CTns have the capacity to transfer between genera, there is a possibility that they may enable both toxigenic and non-toxigenic *C. difficile* strains—as well as other enteric pathogens—to access and incorporate parts of the mitogenome of the intestinal tract. The results also reveal a high abundance of plasmids in *C. difficile*, suggesting a potential role in modulating key physiological functions. Further investigation is necessary to determine how these genetic elements influence the organism’s biology and its capacity for pathogenicity.

## Figures and Tables

**Table 1:** MGEs associated with AMR genes in environmental *C. difficile* strains.

Isolate No.	RT/ST	Tn/IS	AMR Genes
BP201, BP199, BP198, BP197, CF196, CF194, CF193, CF192, CF132, CF129, RSS62, RSS39, RSS64, RSS63, RSS61, RSS66, RSS68, RSS67, CF70, CF72, RSS65, BP71, CF88, CF109, CF90, TDS128, CF89, CF113, CF92	RT127/ST11	Tn*916*	*tetM*
DSS189	RT106/ST42	Tn*916*	*tetM*
		Tn*4001*/IS*256*	*aac(6′)-aph(2″)*
DSS185, DSS184	RT328/ST35	Tn*916*	*tetM*
		Tn*4001*/IS*256*	*aac(6′)-aph(2″)*
		Tn*6073*	
CF107, CF79, ASS23, ASS24, ASS25, ASS26, CF80, CF69, CF75, CF74, CF73, CF77, CF76, CF83, CF81, CF78	RT126/ST11	Tn*916*	*tetM*
DS181, DS159, DS158		Tn*916*	*tetM*
		Tn*6194*-like	*ermB*
DS180, DS179, DS171	RT078/ST11	Tn*916*	*tetM*
DS177, DS155, DS156		Tn*6194*-like	*ermB*
ARE146	RT085/ST39	Tn*916*	*tetM*
RSS37	RT031/ST29	Tn*916*	*tetM*
		Tn*6073*	
DS161, DS157	RT001/ST3	Tn*4001*/IS*256*	*aac(6′)-aph(2″)*
		Tn*6107*	
DS163, RS154, RS153, RS148, TDB131, TDB130, RS17, TDB126, TDS115, TDS123		Tn*6107*	
RS35, ASS20	RT012/ST54	Tn*4001*/IS*256*	*aac(6′)-aph(2″)*
		Tn*5801*-like	*tetM*
RSS10		Tn*4001*/IS*256*	*aac(6′)-aph(2″)*
S45	RT014/ST2	Tn*6073*	
RSS5	RT020/ST2		
TDS118	RT140/ST515	Tn*5397*	*tetM*
		Tn*6110*	
RSS12, RSS52	RT140/ST26	Tn*5397*	*tetM*
		Tn*6110*	
RSS11	RT010/ST15	Tn*6215*	*ermB*

RSS: raw sewage sludge, RS: raw sewage, ASS: activated sewage sludge, DSS: digested sewage sludge, CF: calf feces, BP: biogas plant, ARC: anaerobic lab-scale bioreactors treating sewage sludge/control, ARE: anaerobic lab-scale bioreactors treating sewage sludge/experiment, DS: digested-sewage-sludge-amended soils, TDS: thermophilic digester for treating sewage sludge, TDB: thermophilic digester for treating biowaste.

## Data Availability

All supporting data and protocols have been provided within the article or through Supplementary Data Files.

## Supplementary Materials

The following supporting information can be downloaded at: https://www.mdpi.com/article/10.3390/antibiotics14070678/s1, Table S1: MGEs associated with AMR genes in the genomes of 166 *C. difficile* isolates in this study; Table S2: Potential identified plasmids in the genomes of environmental *C. difficile* strains; Table S3: The occurrence of prophages predicted in identified plasmids of environmental *C. difficile* strains, which carry phage-related genes.

## Abbreviations

The following abbreviations were used in this manuscript:

CDI	*C. difficile* infection
WGS	Whole genome sequencing
CTns	Conjugative transposons
MGEs	Mobile genetic elements
AMR	Antimicrobial resistance
MTns	Mobilizable transposons
MLST	Multilocus sequence typing
IS	Insertion sequence
ICEs	Integrative conjugative elements
HGT	Horizontal gene transfer
MLS_B_	Macrolide–lincosamide–streptogramin B
GI	Genomic island
ST	Sequence type
ORF	Open reading frame
