# Supplementary material for: Mobilome of Environmental Isolates of Clostridioides difficile
Source: Antibiotics (Basel). 2025 Jul 4;14(7):678. doi: 10.3390/antibiotics14070678 (PMC12291638; doi:10.3390/antibiotics14070678)
Supplement: Supplementary file 1 [file antibiotics-14-00678-s001.zip › antibiotics-3722551-supplementary.pdf]

Table S1: MGEs associated with AMR genes in the genomes of 166 *C. difficile* isolates in this study.

Isolate No.	Source	RTs	STs	MLST clade	Toxin genes	Tn/IS	AMR genes
RSS1	RSS	RT005	ST6	1	*tcdA*, *tcdB*		
RSS2		RT090	ST46	1	*tcdA*, *tcdB*		
RSS3		RT011	ST36	1	*tcdA*, *tcdB*		
RSS4		UC	ST6	1	*tcdA*, *tcdB*		
RSS5		RT020	ST2	1	*tcdA*, *tcdB*	Tn*6073*	
RSS6		RT070	ST55	1	*tcdA*, *tcdB*		
RSS7		RT159	ST8	1	*tcdA*, *tcdB*		
RSS10		RT012	ST54	1	*tcdA*, *tcdB*	Tn*4001*/ IS*256*	*aac(6′)-aph(2″)*
RSS11		RT010	ST15	1		Tn*6215*	*ermB*
RSS12		RT140	ST26	1		Tn*5397*	*tetM*
						Tn*6110*	
RSS13		RT023	ST5	3	*tcdA*, *tcdB*, *cdtAB*		
RSS37		RT031	ST29	1		Tn*916*	*tetM*
						Tn*6073*	
RSS38		RT001	ST3	1	*tcdA*, *tcdB*		
RSS39		RT017	ST37	4	*tcdA*, *tcdB*	Tn*916*	*tetM*
RSS52		RT140	ST26	1		Tn*5397*	*tetM*
						Tn*6110*	
RSS61		RT127	ST11	5	*tcdA*, *tcdB*, *cdtAB*	Tn*916*	*tetM*
RSS62		RT127	ST11	5	*tcdA*, *tcdB*, *cdtAB*	Tn*916*	*tetM*
RSS63		RT127	ST11	5	*tcdA*, *tcdB*, *cdtAB*	Tn*916*	*tetM*
RSS64		RT127	ST11	5	*tcdA*, *tcdB*, *cdtAB*	Tn*916*	*tetM*
RSS65		RT127	ST11	5	*tcdA*, *tcdB*, *cdtAB*	Tn*916*	*tetM*
RSS66		RT127	ST11	5	*tcdA*, *tcdB*, *cdtAB*	Tn*916*	*tetM*
RSS67		RT127	ST11	5	*tcdA*, *tcdB*, *cdtAB*	Tn*916*	*tetM*
RSS68		RT127	ST11	5	*tcdA*, *tcdB*, *cdtAB*	Tn*916*	*tetM*
RS8	RS	RT015	ST44	1	*tcdA*, *tcdB*, *cdtAB*		
RS9		UC	ST254	4			
RS14		RT014	ST14	1	*tcdA*, *tcdB*		
RS15		RT023	ST5	3	*tcdA*, *tcdB*, *cdtAB*		
RS16		RT018	ST17	1	*tcdA*, *tcdB*		
RS17		RT001	ST3	1	*tcdA*, *tcdB*	Tn*6107*	
RS32		RT070	ST55	1	*tcdA*, *tcdB*		
RS35		RT012	ST54	1	*tcdA*, *tcdB*	Tn*5801*-like	*tetM*
						Tn*4001*/ IS*256*	*aac(6′)-aph(2″)*
RS36		RT070	ST55	1	*tcdA*, *tcdB*		
RS43		RT011	ST325	1	*tcdA*, *tcdB*		
RS44		RT002	ST8	1	*tcdA*, *tcdB*		
RS147		RT073	ST109	4			
RS148		RT001	ST3	1	*tcdA*, *tcdB*	Tn*6107*	
RS149		RT073	ST109	4			
RS150		RT073	ST109	4			
RS151		RT073	ST109	4			
RS152		RT073	ST109	4			
RS153		RT001	ST3	1	*tcdA*, *tcdB*	Tn*6107*	
RS154		RT001	ST3	1	*tcdA*, *tcdB*	Tn*6107*	
RS164		RT073	ST109	4			
RS165		RT073	ST109	4			
DSS18	DSS	UC	ST8	1	*tcdA*, *tcdB*		
DSS19		UC	ST917	-			
DSS27		RT020	ST2	1	*tcdA*, *tcdB*		
DSS28		RT258	ST58	1	*tcdA*, *tcdB*		
DSS29		RT258	ST58	1	*tcdA*, *tcdB*		
DSS30		RT106	ST42	1	*tcdA*, *tcdB*		
DSS31		RT258	ST58	1	*tcdA*, *tcdB*		
DSS41		RT014	ST13	1	*tcdA*, *tcdB*		
DSS183		RT018	ST17	1	*tcdA*, *tcdB*		
DSS186		RT015	ST44	1	*tcdA*, *tcdB*		
DSS187		RT103	ST53	1	*tcdA*, *tcdB*		
DSS188		RT002	ST8	1	*tcdA*, *tcdB*		

Table S1. *Cont.*

Isolate No.	Source	RTs	STs	MLST clade	Toxin genes	Tn/IS	AMR genes
DSS190	DSS	RT018	ST17	1	*tcdA, tcdB*		
DSS184		RT328	ST35	1	*tcdA, tcdB*	Tn*916*	*tetM*
						Tn*6073*	
						Tn*4001*/ IS*256*	*aac(6′)-aph(2″)*
DSS185		RT328	ST35	1	*tcdA, tcdB*	Tn*916*	*tetM*
						Tn*6073*	
						Tn*4001*/ IS*256*	*aac(6′)-aph(2″)*
DSS189		RT106	ST42	1	*tcdA, tcdB*	Tn*916*	*tetM*
						Tn*4001*/ IS*256*	*aac(6′)-aph(2″)*
DSS191		RT020	ST2	1	*tcdA, tcdB*		
DSS202		UC	ST254	4			
ASS20	ASS	RT012	ST54	1	*tcdA, tcdB*	Tn*5801*-like	*tetM*
						Tn*4001*/ IS*256*	*aac(6′)-aph(2″)*
ASS21		RT014	ST2	1	*tcdA, tcdB*		
ASS22		RT014	ST2	1	*tcdA, tcdB*		
ASS23		RT126	ST11	5	*tcdA, tcdB, cdtAB*	Tn*916* (2)	*tetM*
ASS24		RT126	ST11	5	*tcdA, tcdB, cdtAB*	Tn*916* (2)	*tetM*
ASS25		RT126	ST11	5	*tcdA, tcdB, cdtAB*	Tn*916*	*tetM*
						Tn*916*	*tetM*
ASS26		RT126	ST11	5	*tcdA, tcdB, cdtAB*	Tn*916*	*tetM*
						Tn*916*	*tetM*
S45	Soil	RT014	ST2	1	*tcdA, tcdB*	Tn*6073*	
CF69	CF	RT126	ST11	5	*tcdA, tcdB, cdtAB*	Tn*916*	*tetM*
						Tn*916*	*tetM*
CF70		RT127	ST11	5	*tcdA, tcdB, cdtAB*	Tn*916*	*tetM*
CF72		RT127	ST11	5	*tcdA, tcdB, cdtAB*	Tn*916*	*tetM*
CF73		RT126	ST11	5	*tcdA, tcdB, cdtAB*	Tn*916*	*tetM*
						Tn*916*	*tetM*
CF74		RT126	ST11	5	*tcdA, tcdB, cdtAB*	Tn*916*	*tetM*
						Tn*916*	*tetM*
						Tn*916*	*tetM*
CF75		RT126	ST11	5	*tcdA, tcdB, cdtAB*	Tn*916*	*tetM*
						Tn*916*	*tetM*
CF76		RT126	ST11	5	*tcdA, tcdB, cdtAB*	Tn*916*	*tetM*
						Tn*916*	*tetM*
CF77		RT126	ST11	5	*tcdA, tcdB, cdtAB*	Tn*916*	*tetM*
						Tn*916*	*tetM*
						Tn*916*	*tetM*
CF78		RT126	ST11	5	*tcdA, tcdB, cdtAB*	Tn*916*	*tetM*
						Tn*916*	*tetM*
CF79		RT126	ST11	5	*tcdA, tcdB, cdtAB*	Tn*916*	*tetM*
CF80		RT126	ST11	5	*tcdA, tcdB, cdtAB*	Tn*916*	*tetM*
						Tn*916*	*tetM*
CF81		RT126	ST11	5	*tcdA, tcdB, cdtAB*	Tn*916*	*tetM*
						Tn*916*	*tetM*
						Tn*916*	*tetM*
						Tn*916*	*tetM*
CF82		RT126	ST11	5	*tcdA, tcdB, cdtAB*		
CF83		RT126	ST11	5	*tcdA, tcdB, cdtAB*	Tn*916*	*tetM*
CF84		RT126	ST11	5	*tcdA, tcdB, cdtAB*		
CF85		RT126	ST11	5	*tcdA, tcdB, cdtAB*		
CF86		RT126	ST11	5	*tcdA, tcdB, cdtAB*		
CF87		RT126	ST11	5	*tcdA, tcdB, cdtAB*		
CF88		RT127	ST11	5	*tcdA, tcdB, cdtAB*	Tn*916*	*tetM*
CF89		RT127	ST11	5	*tcdA, tcdB, cdtAB*	Tn*916*	*tetM*
CF90		RT127	ST11	5	*tcdA, tcdB, cdtAB*	Tn*916*	*tetM*
CF92		RT127	ST11	5	*tcdA, tcdB, cdtAB*	Tn*916*	*tetM*
CF95		RT126	ST11	5	*tcdA, tcdB, cdtAB*		
CF97		UC	ST11	5	*tcdA, tcdB, cdtAB*		

Table S1: *Cont.*

Isolate No.	Source	RTs	STs	MLST clade	Toxin genes	Tn/IS	AMR genes
CF99	CF	RT126	ST11	5	*tcdA, tcdB, cdtAB*		
CF101		UC	ST11	5	*tcdA, tcdB, cdtAB*		
CF102		UC	ST11	5	*tcdA, tcdB, cdtAB*		
CF103		UC	ST11	5	*tcdA, tcdB, cdtAB*		
CF107		RT126	ST11	5	*tcdA, tcdB, cdtAB*	Tn*916*	*tetM*
CF109		RT127	ST11	5	*tcdA, tcdB, cdtAB*	Tn*916*	*tetM*
CF113		RT127	ST11	5	*tcdA, tcdB, cdtAB*	Tn*916*	*tetM*
CF114		UC	ST11	5	*tcdA, tcdB, cdtAB*		
CF129		RT127	ST11	5	*tcdA, tcdB, cdtAB*	Tn*916*	*tetM*
CF132		RT127	ST11	5	*tcdA, tcdB, cdtAB*	Tn*916*	*tetM*
CF192		RT127	ST11	5	*tcdA, tcdB, cdtAB*	Tn*916*	*tetM*
CF193		RT127	ST11	5	*tcdA, tcdB, cdtAB*	Tn*916*	*tetM*
CF194		RT127	ST11	5	*tcdA, tcdB, cdtAB*	Tn*916*	*tetM*
CF195		RT127	ST11	5	*tcdA, tcdB, cdtAB*		
CF196		RT127	ST11	5	*tcdA, tcdB, cdtAB*	Tn*916*	*tetM*
CF200		RT126	ST11	5	*tcdA, tcdB, cdtAB*		
BP71	BP	RT127	ST11	5	*tcdA, tcdB, cdtAB*	Tn*916*	*tetM*
BP197		RT127	ST11	5	*tcdA, tcdB, cdtAB*	Tn*916*	*tetM*
BP198		RT127	ST11	5	*tcdA, tcdB, cdtAB*	Tn*916*	*tetM*
BP199		RT127	ST11	5	*tcdA, tcdB, cdtAB*	Tn*916*	*tetM*
BP201		RT127	ST11	5	*tcdA, tcdB, cdtAB*	Tn*916*	*tetM*
TDS115	TDS	RT001	ST3	1	*tcdA, tcdB*	Tn*6107*	
TDS116		RT076	ST2	1	*tcdA, tcdB*		
TDS117		RT014	ST49	1	*tcdA, tcdB*		
TDS118		RT140	ST515	1		Tn*5397*<br>Tn*6110*	*tetM*
TDS119		RT014	ST2	1	*tcdA, tcdB*		
TDS120		RT076	ST2	1	*tcdA, tcdB*		
TDS121		RT076	ST2	1	*tcdA, tcdB*		
TDS122		RT076	ST2	1	*tcdA, tcdB*		
TDS128		RT127	ST11	5	*tcdA, tcdB, cdtAB*	Tn*916*	*tetM*
TDB123	TDB	RT001	ST3	1	*tcdA, tcdB*	Tn*6107*	
TDB124		RT023	ST5	3	*tcdA, tcdB, cdtAB*		
TDB126		RT001	ST3	1	*tcdA, tcdB*	Tn*6107*	
TDB127		RT023	ST5	3	*tcdA, tcdB, cdtAB*		
TDB130		RT001	ST3	1	*tcdA, tcdB*	Tn*6107*	
TDB131		RT001	ST3	1	*tcdA, tcdB*	Tn*6107*	
ARC134	ARC	RT095	ST2	1	*tcdA, tcdB*		
ARC135		RT077	ST13	1	*tcdA, tcdB*		
ARC139		RT015	ST44	1	*tcdA, tcdB*		
ARC140		RT014	ST2	1	*tcdA, tcdB*		
ARC141		RT020	ST2	1	*tcdA, tcdB*		
ARC167		UC	ST8	1	*tcdA, tcdB*		
ARC168		RT159	ST8	1	*tcdA, tcdB*		
ARC182		RT005	ST6	1	*tcdA, tcdB*		
ARE136	ARE	RT120	ST4	1	*tcdA, tcdB*		
ARE137		RT120	ST4	1	*tcdA, tcdB*		
ARE138		RT120	ST4	1	*tcdA, tcdB*		
ARE143		RT120	ST4	1	*tcdA, tcdB*		
ARE144		RT120	ST4	1	*tcdA, tcdB*		
ARE145		UC	ST109	4			
ARE146		RT085	ST39	4	*tcdA, tcdB*	Tn*916*	*tetM*
ARE170		RT120	ST4	1	*tcdA, tcdB*		
DS155	DSS-S	RT078	ST11	5	*tcdA, tcdB, cdtAB*	Tn*6194*-like	*ermB*
DS156		RT078	ST11	5	*tcdA, tcdB, cdtAB*	Tn*6194*-like	*ermB*
DS157		RT001	ST3	1	*tcdA, tcdB*	Tn*6107*<br>Tn*4001*/ IS*256*	 *aac(6′)-aph(2″)*
DS172		UC	UN	4			
DS173		RT078	ST11	5	*tcdA, tcdB, cdtAB*		

Table S1: *Cont.*

Isolate No.	Source	RTs	STs	MLST clade	Toxin genes	Tn/IS	AMR genes
DS174	DSS-S	RT078	ST11	5	*tcdA, tcdB, cdtAB*		
DS175		RT078	ST11	5	*tcdA, tcdB, cdtAB*		
DS176		RT078	ST11	5	*tcdA, tcdB, cdtAB*		
DS177		RT078	ST11	5	*tcdA, tcdB, cdtAB*	Tn*6194*-like	*ermB*
DS178		RT120	ST4	1	*tcdA, tcdB, cdtAB*		
DS158		RT126	ST11	5	*tcdA, tcdB, cdtAB*	Tn*6194*-like	*ermB*
						Tn*916*	*tetM*
						Tn*916*	*tetM*
DS159		RT126	ST11	5	*tcdA, tcdB, cdtAB*	Tn*6194*-like	*ermB*
						Tn*916* (2)	*tetM*
DS160		UC	ST124	4			
DS161		RT001	ST3	1	*tcdA, tcdB*	Tn*6107*	
						Tn*4001*/ IS*256*	*aac(6′)-aph(2″)*
DS162		RT078	ST11	5	*tcdA, tcdB, cdtAB*		
DS163		RT001	ST3	1	*tcdA, tcdB*	Tn*6107*	
DS169		UC	ST821	-			
DS171		RT078	ST11	5	*tcdA, tcdB, cdtAB*	Tn*916*	*tetM*
DS179		RT078	ST11	5	*tcdA, tcdB, cdtAB*	Tn*916*	*tetM*
DS180		RT078	ST11	5	*tcdA, tcdB, cdtAB*	Tn*916*	*tetM*
DS181		RT126	ST11	5	*tcdA, tcdB, cdtAB*	Tn*6194*-like	*ermB*
						Tn*916* (2)	*tetM*

RSS: raw sewage sludge, RS: raw sewage, ASS: activated sewage sludge, DSS: digested sewage sludge, CF: calf feces, BP: biogas plant, ARC: anaerobic lab-scale bioreactors treating sewage sludge/control, ARE: anaerobic lab-scale bioreactors treating sewage sludge/experiment, DS: digested sewage sludge-amended soils, TDS: thermophilic digester for treating sewage sludge, TDB: thermophilic digester for treating biowaste.

Table S2: Potential identified plasmids in the genomes of environmental *C. difficile* strains.

Strain No.	Accession No.	RT/ST	No. of plasmids	Identified plasmids	Size (bp)	Reference plasmid in NCBI					Predicted prophages
						Organism	Plasmid	Accession No.	Coverage (%)	Identity (%)	
DSS190	JAVJBD010000006.1	RT018/ST17	1	pDSS190_6	12,486	*C. difficile*	pAR1088-1	CP126073.1	100	99.97	
DSS191	JAVJBC010000003.1	RT020/ST2	2	pDSS191_3	13,652	*C. difficile*	pCD6	AY350745.1	99	100	
	JAVJBC010000004.1			pDSS191_4	12,333	*C. difficile*	pCD6	AY350745.1	99	100	
ARE137	JAVJCZ010000003.1	RT120/ST4	16	pARE137_3	12,333	*C. difficile*	pCd1_3	CP037846.1	100	100	
	JAVJCZ010000004.1			pARE137_4	12,397	*C. difficile*	pCd1_3	CP037846.1	100	100	
	JAVJCZ010000005.1			pARE137_5	12,049	*C. difficile*	pCd7_4	CP037838.1	99	99.97	
	JAVJCZ010000006.1			pARE137_6	10,384	*C. difficile*	pCD6	AY350745.1	99	100	
	JAVJCZ010000007.1			pARE137_7	9,854	*C. difficile*	pCd5_4	CP037843.1	99	100	
	JAVJCZ010000008.1			pARE137_8	9,426	*C. difficile*	pCd5_4	CP037843.1	100	100	
	JAVJCZ010000009.1			pARE137_9	9,415	*C. difficile*	pCd7_4	CP037838.1	100	100	
	JAVJCZ0100000010.1			pARE137_10	9,245	*C. difficile*	pCD6	AY350745.1	100	100	
	JAVJCZ0100000011.1			pARE137_11	8,405	*C. difficile*	pCD6	AY350745.1	100	99.26	
	JAVJCZ0100000012.1			pARE137_12	8,399	*C. difficile*	pCd5_4	CP037843.1	100	100	
	JAVJCZ0100000013.1			pARE137_13	8,023	*C. difficile*	pCD6	AY350745.1	100	100	
	JAVJCZ0100000014.1			pARE137_14	7,282	*C. difficile*	pCd7_4	CP037838.1	100	99.85	
	JAVJCZ0100000015.1			pARE137_15	7,789	*C. difficile*	pCd1_3	CP037846.1	100	100	
	JAVJCZ0100000016.1			pARE137_16	7,539	*C. difficile*	pCD6	AY350745.1	100	100	
	JAVJCZ0100000017.1			pARE137_17	7,255	*C. difficile*	pCD6	AY350745.1	99	100	
	JAVJCZ0100000018.1			pARE137_18	7,115	*C. difficile*	pCD6	AY350745.1	99	100	
DSS188	JAVJBE010000003.1	RT002/ST8	3	pDSS188_3	12,523	*C. difficile*	pCd11_4	CP037825.1	100	99.98	
	JAVJBE010000005.1			pDSS188_5	4,674	*C. difficile*	pCD-ECE6	LR594546.1	100	99.97	
	JAVJBE010000007.1			pDSS188_7	46,312	*C. difficile*	p830101_1	CP132145.1	92	100	phiCDHM19
DSS186	JAVJBG010000003.1	RT015/ST44	5	pDSS186_3	24,433	*C. difficile*	pCD-WTSI2	MG019960.1	100	100	
	JAVJBG010000004.1			pDSS186_4	12,757	*C. difficile*	pCd1_3	CP037846.1	99	99.99	
	JAVJBG010000005.1			pDSS186_5	9,061	*C. difficile*	pCd1_3	CP037846.1	100	100	
	JAVJBG010000008.1			pDSS186_8	6,417	*C. difficile*	pCd8	CP037834.1	100	100	
	JAVJBG010000009.1			pDSS186_9	12,525	*C. difficile*	unnamed3 plasmid	CP083619.1	100	100	
DSS187	JAVJBF010000006.1	RT103/ST53	2	pDSS187_6	20,663	*C. difficile*	pCd11_5	CP037826.1	99	99.99	
	JAVJBF010000007.1			pDSS187_7	7,796	*C. difficile*	pCd11_5	CP037826.1	100	99.80	
DSS183	JAVJBJ010000005.1	RT018/ST17	1	pDSS183_5	12,488	*C. difficile*	pAR1088-1	CP126073.1	99	100	
DSS27	JAVJFS010000002.1	RT020/ST2	2	pDSS27_2	15,627	*C. difficile*	pCd5_4	CP037843.1	100	99.98	
	JAVJFS010000003.1			pDSS27_3	7,969	*C. difficile*	pCD6	AY350745.1	100	100	
DSS29	JAVJFQ010000003.1	RT258/ST58	3	pDSS29_3	22,150	*C. difficile*	pAR1088-1	CP126073.1	95	99.98	
	JAVJFQ010000004.1			pDSS29_4	13,369	*C. difficile*	pCD-WTSI1	MG019959.1	99	99.70	
	JAVJFQ010000005.1			pDSS29_5	6,853	*C. difficile*	pCD-ECE4	LR594545.1	100	99.98	
ARE143	JAVJCU010000006.1	RT120/ST4	1	pARE143_6	10,632	*C. difficile*	pCD6	AY350745.1	100	100	

Table S2. *Cont.*

Strain No.	Accession No.	RT/ST	No. of plasmids	Identified plasmids	Size (bp)	Reference plasmid in NCBI					Predicted prophages
						Organism	Plasmid	Accession No.	Coverage (%)	Identity (%)	
DSS31	JAVJFO010000004.1	RT258/ST58	2	pDSS31_4	11,882	*C. difficile*	pCD-WTSI1	MG019959.1	99	99.70	
	JAVJFO010000005.1			pDSS31_5	6,850	*C. difficile*	pCD-ECE4	LR594545.1	100	99.98	
DSS28	JAVJFR010000002.1	RT258/ST58	2	pDSS28_2	6,856	*C. difficile*	pCD-ECE4	LR594545.1	100	99.98	
	JAVJFR010000003.1			pDSS28_3	11,881	*C. difficile*	pCD-WTSI1	MG019959.1	99	99.70	
ARE170	JAVJBU010000004.1	RT120/ST4	2	pARE170_4	28,589	*C. difficile*	pCD6	AY350745.1	100	99.97	
	JAVJBU010000006.1			pARE170_6	12,928	*C. difficile*	pCD6	AY350745.1	100	99.97	
ARE138	JAVJCY010000004.1	RT120/ST4	4	pARE138_4	13,365	*C. difficile*	pCd5_4	CP037843.1	100	100	
	JAVJCY010000005.1			pARE138_5	13,630	*C. difficile*	pCd5_4	CP037843.1	100	100	
	JAVJCY010000006.1			pARE138_6	10,465	*C. difficile*	pCd1_3	CP037846.1	100	100	
	JAVJCY010000007.1			pARE138_7	7,390	*C. difficile*	pCD6	AY350745.1	100	100	
ARE144	JAVJCT010000004.1	RT120/ST4	2	pARE144_4	11,566	*C. difficile*	pCD6	AY350745.1	100	100	
	JAVJCT010000005.1			pARE144_5	7,001	*C. difficile*	pCD6	AY350745.1	100	100	
ARE136	JAVJDA010000005.1	RT120/ST4	5	pARE136_5	12,812	*C. difficile*	pCd1_3	CP037846.1	100	100	
	JAVJDA010000006.1			pARE136_6	7,317	*C. difficile*	pCD6	AY350745.1	100	100	
	JAVJDA010000007.1			pARE136_7	6,929	*C. difficile*	pCD6	AY350745.1	100	100	
	JAVJDA010000008.1			pARE136_8	6,767	*C. difficile*	pCD6	AY350745.1	100	100	
	JAVJDA0100000010.1			pARE136_10	6,830	*C. difficile*	pCd7_4	CP037838.1	100	100	
CF82	JAVJEI010000006.1	RT126/ST11	1	pCF82_6	5,262	*Escherichia coli*	pB1.H1.1.c6	CP146568.1	99	97.28	
RSS1	JAVJGF010000003.1	RT005/ST6	2	pRSS1_3	48,344	*C. difficile*	pCd10_2	CP037828.1	92	98.56	phiCDHM19
	JAVJGF010000007.1			pRSS1_7	12,526	*C. difficile*	pCD-WTSI2	MG019960.1	100	99.99	
RSS4	JAVJGE010000003.1	UC/ST6	2	pRSS4_3	48,344	*C. difficile*	pCd10_2	CP037828.1	92	98.56	phiCDHM19
	JAVJGE010000006.1			pRSS4_6	12,526	*C. difficile*	pCd10_2	CP037828.1	100	97.64	
RSS7	JAVJGC010000004.1	RT159/ST8	2	pRSS7_4	46,315	*C. difficile*	pJ21_1	CP134691.1	99	99.99	phiCDHM19
	JAVJGC010000006.1			pRSS7_6	17,223	*C. difficile*	pCD6	AY350745.1	99	99.98	
RSS6	JAVJGD010000004.1	RT070/ST55	1	pRSS6_4	4,690	*C. difficile*	pAR1090-2	CP126070.1	100	100	
RS9	JAVJGA010000002.1	UC/ST254	2	pRS9_2	120,098	*Clostridioides sp.*	pESS17301a	CP067354.1	82	92.38	
	JAVJGA010000007.1			pRS9_7	801	*Enterococcus faecium*	plasmid P1	CP111427.1	100	99.63	
RS8	JAVJGB010000002.1	RT015/ST44	5	pRS8_2	47,964	*C. difficile*	pCd10_2	CP037828.1	88	96.39	phiCDHM19
	JAVJGB010000003.1			pRS8_3	12,526	*C. difficile*	pCD-WTSI2	MG019960.1	100	100	
	JAVJGB010000004.1			pRS8_4	6,853	*C. difficile*	pCD-ECE4	LR594545.1	100	100	
	JAVJGB010000005.1			pRS8_5	4,675	*C. difficile*	pCD-ECE6	LR594546.1	100	100	
	JAVJGB010000006.1			pRS8_6	797	*E. faecium*	p15-307-1_05	CP044321.1	100	98.50	
RS15	JAVJFX010000002.1	RT023/ST5	1	pRS15_2	4,675	*C. difficile*	pCD-ECE6	LR594546.1	100	100	
TDS117	JAVJDS010000004.1	RT014/ST49	2	pTDS117_4	12,508	*C. difficile*	pCD-WTSI4	MG019962.1	100	100	
	JAVJDS010000005.1			pTDS117_5	9,177	*C. difficile*	pAR1090-2	CP126070.1	100	100	
RS14	JAVJFY010000002.1	RT014/ST14	3	pRS14_2	12,488	*C. difficile*	pCD-WTSI4	MG019962.1	100	99.99	

Table S2. *Cont.*

Strain No.	Accession No.	RT/ST	No. of plasmids	Plasmids	Size (bp)	Reference plasmid in NCBI					Predicted prophages
						Organism	Plasmid	Accession No.	Coverage (%)	Identity (%)	
RS14	JAVJFY010000003.1	RT014/ST14	3	pRS14_3	6,257	*C. difficile*	pCD-ECE5	LR594541.1	100	99.94	
	JAVJFY010000004.1			pRS14_4	4,675	*C. difficile*	pCD-ECE6	LR594546.1	100	100	
RSS39	JAVJFJ010000003.1	RT017/ST37	1	pRSS39_3	48,590	*C. difficile*	unnamed1 plasmid	CP029153.1	100	99.99	phiCDHM19
RS44	JAVJFG010000005.1	RT002/ST8	5	pRS44_5	20,534	*C. difficile*	pCd1_3	CP037846.1	100	99.74	
	JAVJFG010000006.1			pRS44_6	9,103	*C. difficile*	pCD6	AY350745.1	100	100	
	JAVJFG010000007.1			pRS44_7	8,254	*C. difficile*	pCD6	AY350745.1	100	100	
	JAVJFG010000009.1			pRS44_9	12,486	*C. difficile*	pCD-WTSI4	MG019962.1	100	100	
	JAVJFG0100000011.1			pRS44_11	46,315	*C. difficile*	pJ21_1	CP134691.1	99	99.99	phiCDHM19
TDB123	JAVJDO010000002.1	RT001/ST3	2	pTDB123_2	45,290	*C. difficile*	pCd11_3	CP037824.1	97	99.64	phiCDHM19
	JAVJDO010000006.1			pTDB123_6	12,544	*C. difficile*	pCd28_4	CP037800.1	99	99.95	
TDB127	JAVJDL010000009.1	RT023/ST5	1	pTDB127_9	8,958	*C. difficile*	pAR1090-2	CP126070.1	100	100	
TDB126	JAVJDM010000008.1	RT001/ST3	2	pTDB126_8	12,527	*C. difficile*	pCd14_2	CP037819.1	100	100	
	JAVJDM010000009.1			pTDB126_9	45,285	*C. difficile*	pCd11_3	CP037824.1	97	99.66	phiCDHM19
TDB130	JAVJDI010000004.1	RT001/ST3	2	pTDB130_4	12,560	*C. difficile*	pCD-WTSI2	MG019960.1	99	99.95	
	JAVJDI010000005.1			pTDB130_5	45,292	*C. difficile*	pCDBI1	FN668942.1	95	99.73	phiCDHM19
TDS115	JAVJDT010000007.1	RT001/ST3	1	pTDS115_7	45,287	*C. difficile*	pCDBI1	FN668942.1	94	99.92	phiCDHM19
TDB131	JAVJDH010000005.1	RT001/ST3	2	pTDB131_5	12,530	*C. difficile*	pCD-WTSI2	MG019960.1	100	100	
	JAVJDH010000009.1			pTDB131_9	45,280	*C. difficile*	pCDBI1	FN668942.1	95	99.75	phiCDHM19
DS178	JAVJBN010000006.1	RT120/ST4	3	pDS178_6	14,707	*C. difficile*	pCd8_4	CP037834.1	99	100	
	JAVJBN0100000010.1			pDS178_10	6,865	*C. difficile*	pCd1_3	CP037846.1	100	100	
	JAVJBN0100000011.1			pDS178_11	13,540	*C. difficile*	pCd11_5	CP037826.1	100	100	
DS163	JAVJCA010000004.1	RT001/ST3	1	pDS163_4	45,285	*C. difficile*	pCDBI1	FN668942.1	94	99.75	phiCDHM19
ARC141	JAVJCV010000002.1	RT020/ST2	3	pARC141_2	13,260	*C. difficile*	pCD6	AY350745.1	100	100	
	JAVJCV010000003.1			pARC141_3	7,220	*C. difficile*	pCD6	AY350745.1	100	100	
	JAVJCV010000004.1			pARC141_4	6,826	*C. difficile*	pCD6	AY350745.1	99	99.93	
ARC139	JAVJCX010000006.1	RT015/ST44	2	pARC139_6	47,972	*C. difficile*	pCd10_2	CP037828.1	88	96.76	phiCDHM19
	JAVJCX010000007.1			pARC139_7	12,541	*C. difficile*	pCd11_4	CP037825.1	100	100	
ARC167	JAVJBX010000002.1	UC/ST8	2	pARC167_2	12,528	*C. difficile*	pCd8_3	CP037833.1	100	99.96	
	JAVJBX010000003.1			pARC167_3	46,314	*C. difficile*	p830101_1	CP132145.1	92	99.99	phiCDHM19
ARC182	JAVJBK010000004.1	RT005/ST6	4	pARC182_4	25,248	*C. difficile*	pCd10_2	CP037828.1	86	99.98	phiCDHM11
	JAVJBK010000005.1			pARC182_5	12,526	*C. difficile*	pCd28_4	CP037800.1	100	99.95	
	JAVJBK010000006.1			pARC182_6	6,760	*C. difficile*	pCD6	AY350745.1	100	100	
	JAVJBK010000007.1			pARC182_7	48,278	*C. difficile*	p830101_1	CP132145.1	92	100	phiCDHM19
ARC168	JAVJBW010000004.1	RT159/ST8	2	pARC168_4	4,676	*C. difficile*	pCD-ECE6	LR594546.1	100	100	
	JAVJBW010000006.1			pARC168_6	46,306	*C. difficile*	p830101_1	CP132145.1	92	100	phiCDHM19

RSS: raw sewage sludge, RS: raw sewage, DSS: digested sewage sludge, CF: calf feces, ARC: anaerobic lab-scale bioreactors treating sewage sludge/control, ARE: anaerobic lab-scale bioreactors treating sewage sludge/experiment, DS: digested sewage sludge-amended soils, TDS: thermophilic digester for treating sewage sludge, TDB: thermophilic digester for treating biowaste.

Table S3. The occurrence of prophages predicted in identified plasmids of environmental *C. difficile* strains, which carry phage-related genes.

Plasmid No.	Region	Region length (kb)	Completeness	Score	#Total protein	Region position	Most Common Phage	GC%
DSS188_7	1	46.3	Intact	150	72	2-46312	PHAGE_Clostr_phiCDHM19_NC_028996(21)	28.31
RSS1_3	1	46.9	Intact	150	65	1146-48136	PHAGE_Clostr_phiCDHM19_NC_028996(21)	28.26
RSS4_3	1	47.8	Intact	150	68	2-47879	PHAGE_Clostr_phiCDHM19_NC_028996(21)	28.28
RSS7_4	1	31.1	Intact	140	51	1-31192	PHAGE_Clostr_phiCDHM19_NC_028996(12)	28.20
	2	13.4	Incomplete	50	18	32847-46315	PHAGE_Clostr_phiCDHM19_NC_028996(9)	28.85
RS8_2	1	47.9	Intact	150	77	3-47963	PHAGE_Clostr_phiCDHM19_NC_028996(21)	28.44
RSS39_3	1	37	Intact	150	55	11519-48590	PHAGE_Clostr_phiCDHM19_NC_028996(18)	29.14
RS44_11	1	30.1	Intact	150	52	1-30103	PHAGE_Clostr_phiCDHM19_NC_028996(12)	28.11
	2	14.5	Incomplete	50	18	31758-46315	PHAGE_Clostr_phiCDHM19_NC_028996(9)	29.00
TDB123_2	1	40.7	Intact	150	44	4395-45114	PHAGE_Clostr_phiCDHM19_NC_028996(17)	28.07
TDB130_5	1	44.8	Intact	150	64	1-44874	PHAGE_Clostr_phiCDHM19_NC_028996(19)	28.13
TDB126_9	1	45.1	Intact	150	69	118-45283	PHAGE_Clostr_phiCDHM19_NC_028996(18)	28.07
TDS115_7	1	40	Intact	150	62	849-40858	PHAGE_Clostr_phiCDHM19_NC_028996(19)	28.60
TDB131_9	1	39.4	Intact	150	57	47-39545	PHAGE_Clostr_phiCDHM19_NC_028996(19)	28.41
DS163_4	1	39.8	Intact	150	57	2-39868	PHAGE_Clostr_phiCDHM19_NC_028996(19)	28.43
ARC139_6	1	42.3	Intact	150	46	131-42477	PHAGE_Clostr_phiCDHM19_NC_028996(18)	28.51
ARC167_3	1	44.9	Intact	150	70	505-45486	PHAGE_Clostr_phiCDHM19_NC_028996(21)	28.31
ARC182_4	1	13.1	Incomplete	50	20	1-13185	PHAGE_Clostr_phiCDHM11_NC_029001(10)	29.55
ARC182_7	1	46.9	Intact	150	66	1145-48069	PHAGE_Clostr_phiCDHM19_NC_028996(21)	28.29
ARC168_6	1	38	Intact	150	44	8263-46263	PHAGE_Clostr_phiCDHM19_NC_028996(19)	28.34
